# Treatment Outcomes and Associated Factors among Hospitalized Stroke Patients at Shashemene Referral Hospital, Ethiopia

**DOI:** 10.1155/2018/8079578

**Published:** 2018-08-28

**Authors:** Tegegne Gobezie Temesgen, Berhanu Teshome, Peter Njogu

**Affiliations:** ^1^Addis Ababa University, College of Health Sciences, School of Pharmacy, Department of Pharmacology and Clinical Pharmacy, Ethiopia; ^2^Ambo University, College of Medicine and Health Sciences, Department of Pharmacy, Ethiopia; ^3^University of Nairobi, College of Health Sciences, School of Pharmacy, Department of Pharmaceutical Chemistry, Nairobi 19676-00202, Kenya

## Abstract

**Background:**

The 2013 Global Burden of Disease report indicated that 80% of stroke deaths occur in low- and middle-income regions. Although stroke has been consistently reported as one of the three leading causes of morbidity and mortality in the past years in Ethiopia, there is a paucity of data regarding treatment outcomes of stroke if sufficient. Hence, the present study aimed to assess patterns of treatment outcomes and associated factors among hospitalized stroke patients at Shashemene Referral Hospital.

**Methods:**

A retrospective cross-sectional study was conducted at the medical ward of Shashemene Referral Hospital. A total of 73 hospitalized stroke patients during the period 2012–2017 were included in the study. Demographic characteristics, risk factors, and stroke types and their hospital outcomes were reviewed from the medical records of the patients. The data were entered and analyzed using SPSS version 16.0. Descriptive statistics such as percent and frequency were used to summarize patients' characteristics. Binary logistic regression was used to investigate the potential predictors of treatment outcome. A p-value ≤0.05 was considered statistically significant.

**Result:**

Ischemic stroke was the most common type of stroke (65.8%) diagnosed in our setting. Hypertension (52.05%) was the common comorbid condition. More than half (54.79%) of the stroke patients improved on treatment. Dyslipidemics were prescribed to 68.49% of patients and the most popular antiplatelet was aspirin, which was prescribed to 61.64% of the study participants. Age, sex, type of stroke, and type of comorbidity were not significant factors of stroke treatment outcome.

**Conclusion:**

Ischemic stroke was the most common type of stroke diagnosed among the study participants while aspirin and statins were the most frequently used drugs in the management of stroke. Approximately 50% of hospitalized stroke patients had good treatment outcome and none of the investigated variables were significantly associated with the treatment outcomes.

## 1. Background

According to the World Health Organization (WHO), stroke is defined as rapidly developing clinical signs of focal (or global) disturbance of cerebral function, with symptoms lasting 24 hours or longer or leading to death which may be either ischemic or hemorrhagic disturbances of the cerebral blood circulation [1]. In Caucasian populations, approximately 80% of all strokes are ischemic while about 20% are hemorrhagic secondary to intracerebral and subarachnoid hemorrhage [2]. The 2013 Global Burden of Disease study reported that cerebrovascular diseases ranked the second leading cause of death after ischemic heart disease [3]. The WHO has estimated that 15 million people suffer from stroke each year globally with African countries accounting for 86% of the stroke deaths [4, 5]. According to the WHO data published in 2014, stroke accounted for 28,320 (4.71 %) of total deaths in Ethiopia [6] which is consistent with observations of a retrospective study conducted at Tikur Anbessa Specialized Hospital which indicated that stroke accounted for 5% of all head computed tomography (CT) scan indications [7]. Despite the high prevalence of stroke in Ethiopia, there is a paucity of data regarding treatment outcomes and other contributing factors in stroke patients. This study therefore aimed to assess the factors and treatment outcomes of hospitalized stroke patients at Shashemene Referral Hospital.

## 2. Methods

### 2.1. Study Design and Setting

A retrospective cross-sectional study was conducted on hospitalized stroke patients in the medical ward of Shashemene Referral Hospital from March to April 2017. Shashemene Referral Hospital is located in Kuyera town about 238 km south of the capital city Addis Ababa, Ethiopia. It is one of the oldest hospitals of Oromia region serving approximately 2.1 million people.

### 2.2. Eligibility Criteria

Patients who had complete medical records, confirmed diagnosis of stroke, and admitted in the medical ward of the hospital during the period of March 1, 2012–March 31, 2017, were included in the study.

### 2.3. Sampling Method and Sample Size Determination

All patient cards that fulfilled the inclusion criteria were included in the study. Hence, a total of 73 stroke patients who had been hospitalized during the five years (2012–2017) study period were used.

### 2.4. Data Collection Techniques and Procedure

Two qualified nurses from the medical ward of the Hospital were trained to assist in data collection. Identification card number of hospitalized stroke patients who had been hospitalized during the study period was collected from discharge summary of patients. Using this identification card, all relevant information about each patient such as sociodemographic characteristics, clinical information, treatment regimens, and outcomes was recorded by reviewing medical records of the patients. A pretest was done on five patient cards to ensure the validity and reliability of the data collection instruments. After pretesting, all necessary adjustments were employed on the data collection instruments before implementing in the main study.

All sociodemographic and clinical data (including diagnosis and treatment outcome) were obtained from patients' cards and the discharge notes.

Good treatment outcome implies that the patient is discharged without any complication secondary to the stroke attack.

### 2.5. Data Analysis

The collected data were cleaned, entered, processed, and analyzed by SPSS version 16.0 statistical software. Descriptive statistics such as percent and frequency were used to summarize categorical variables of patients' characteristics. Binary logistic regression analyses were employed to investigate the potential predictors of treatment outcomes of hospitalized stroke patients. A p-value ≤0.05 was considered statistically significant.

## 3. Results

### 3.1. Sociodemographic and Clinical Characteristics

A total of 73 stroke patients were included in the study, comprising 42 (57.5%) males and 31 (42.5%) females. Majority of the study participants were married (89%) and most were farmers (41.1%). The mean age and mean length of hospital stay of the patients were 63.2±14.8 years and 6.7±2.5 days, respectively, while the mean time from symptoms onset to hospital admission was 23.50 ±13.14 h. During admission, left-sided body weakness (39.7%) was the most frequently encountered chief complaint among the hospitalized stroke patients. Hypertension (38, 52.05%) was the major c-morbidity while atrial fibrillation was the least common (4.1%) among study participants. Ischemic stroke was the most frequently (65.8%) diagnosed type of stroke among the study participants followed by hemorrhagic stroke (21.9%) as shown in Table 1. This observation is consistent with a study finding in India by Nandigam et al. [8].

### 3.2. Treatment Regimen and Outcome of Hospitalized Stroke Patients

Up to two-thirds of patients (61.6%) used aspirin only while comparable number of patients (59.6%) used both aspirin and statin during admission for stroke management. On discharge, 46.5 % of them used aspirin only while 46.6% used both aspirin and statin. About three-fourths of patients were discharged with aspirin with or without a statin (Table 2).

Nifedipine (24.7%) and enalapril (20.5%) were the commonly used concomitant medications prescribed in the management of comorbidities while hydrochlorothiazide and captopril were among the least frequent concurrently prescribed medications (Table 3).

**Figure 1:**
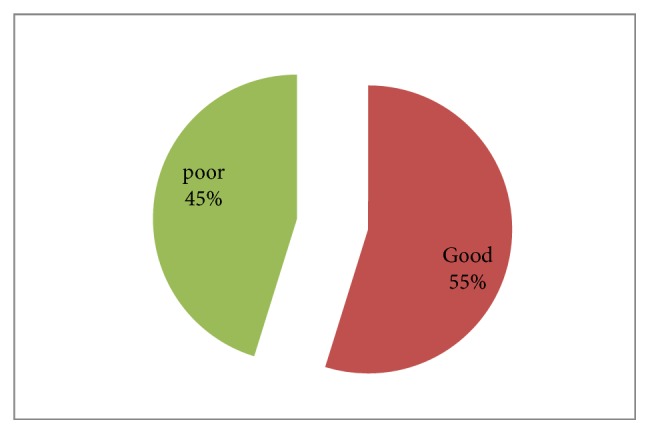
Treatment outcomes of hospitalized stroke patients at Shashemene Referral Hospital, Ethiopia, 2012–2017.

More than half (54.8%) of hospitalized stroke patients had good treatment outcome (Figure 1).

Binary logistic regression analysis revealed that chief complaint, comorbid diseases, type of stroke, and other sociodemographic characteristics did not significantly affect treatment outcome of stroke patients (Table 1).

## 4. Discussion

Approximately half of study participants had successful treatment outcome, while none of the independent variable showed significant association with it. The present study showed that male patients were more prone to stroke than females, an observation that is in line with similar study done by Gedefa et al. [9] at Saint Paul's Teaching Hospital. The mean age of the study participants was 63.16±14.81 years, a finding that is in agreement with a similar study conducted in India [10]. The high prevalence of stroke in older patients could probably be due to the presence of different cardiovascular comorbidities secondary to aging which are potential risk factors for stroke. Besides, majority (53.05%) of the study participants had hypertension, the major risk factor for stroke, as a comorbidity which is commonly associated with aging.

Ischemic stroke (48, 65.8%) was the most prevalent type of stroke in this study, which is lower than a study done in Northwest Ethiopia (69.4%) [11]. On the contrary, similar study done at St. Paul's Teaching Hospital showed hemorrhagic stroke was the most common type of stroke accounting for 61.3% of cases with majority of patients being in the 56–70 years age group [9]. Contrastingly, a previous study by Gedefa et al. [9] showed that the mode age group of the stroke patients was 64–84 years.

In the present study, there was a preponderance of patients who experienced left-sided body weakness (39.7%) as a chief complaint which is relatively higher than but comparable to the reported finding of a study done in Ghana (35%) [12]. Similarly, Garbusinski et al. reported that 41.89% patients had complained of left-sided body weakness during hospital admission [13] while a study by Gedefa et al. showed that the majority (81.6%) of patients presented with focal neurologic deficit [9].

The median time from stroke symptoms onset to hospital admission was 23.50±13.14 h in our setting. In contrast, a Hospital-Based Multicenter Prospective Study in Brazil showed that the median time from symptoms onset to hospital admission was 12.9 h [14]. This disparity may be due to better infrastructure and greater awareness of people about the advantage of early hospital admission in Brazil. A study at University of Gondar Hospital showed that the mean length of hospital stay of stroke patients was 13 days [7].

Regarding the pharmacological management of stroke, 59.6% patients were on lipid lowering medications, of which 38.35% patients used simvastatin. Previous study done in India by Abbasi et al. reported that simvastatin was used in 21.56% patients [15]. This may be due to the availability of other medications used for treatment of stroke in India. Aspirin (45, 61.6%) was the only antiplatelet used in our setting during admission.

A majority of the patients (54.8 %) had good treatment outcome while 15.1% of the patients ended up with various complications. The finding is similar to a study done in North West Ethiopia where 59.18% were discharged with significant improvement and only 13.3% of stroke patients died in the hospital [9]. In contrast, a Germany study showed the overall hospital mortality among hospitalized stroke patients was only 4.9% [16]. This disparity might be attributed to the availability of different pharmacological treatment regimens and better healthcare systems in Germany.

According to binary logistic regression analysis, chief complaint, comorbid diseases, type of stroke, and other sociodemographic characteristics did not significantly affect treatment outcome of stroke patients. Contrary to the finding of this study, advanced age was significantly associated with treatment outcomes in a study conducted in Switzerland [17].

## 5. Conclusion

The present study showed that ischemic stroke was the most common type of stroke diagnosed among the study participants. Aspirin and statins (lovastatin and simvastatin) were the most frequently used drugs in the management of stroke. Around half of hospitalized stroke patients had good treatment outcome and none of the variables were significantly associated with the treatment outcomes.

## Figures and Tables

**Table 1:** Sociodemographic and clinical characteristics of hospitalized stroke patients at Shashemene Referral Hospital, 2012–2017 (N=73).

**Variables**			**Bivariate analysis**		**Multivariate analysis**	
		N (%)	COR	P	AOR	P
**Age**						
	25-40	7(9.6)	0.27	0.112	1.10	0.922
	41-55	11(15.1)	0.27	0.079	0.43	0.374
	56-70	29(39.7)	0.33	0.054	0.41	0.281
	>70	26(35.6)	1.00		1.00	
**Chief compliant**						
	Left side body weakness	29(39.7)	1.00	1.000	1.000	1.000
	Right side body weakness	16(21.9)	1.00	1.000	1.000	1.000
	Aphasia	18(24.7)	1.00	1.000	1.000	1.000
	Loss of consciousness	10(13.7)	1.00		1.00	
**Type of stroke**						
	Ischemic	48(65.8)	1.00		1.00	
	Hemorrhagic	16(21.9)	1.56	0.669	3.77	0.237
	Undetermined	9(12.3)	3.00	0.178	1.62	1.000
**Sex**	Male	42(57.5)	1.00	0.995		
	Female	31(42.5)	0.82	0.591		
**Marital status**						
	Married	65(89)	1.71	0.666		
	Unmarried	5(6.8)	1.33	0.851		
	Widow	3(4.1)	1.00			
**Occupation**						
	Farmer	30(41.1)	0.15	0.102		
	Housewife	21(28.8)	0.15	0.120		
	Civil servant	17(23.3)	0.14	0.399		
	Unemployed	5(6.8)	1.00			
**Co morbidities**						
	Hypertension	38(52.05)	1.00			
	Concurrent infection	23(31.5)	1.54	0.739		
	Congestive heart failure	9(12.32)	2.50	0.512		
	Atrial fibrillation	3(4.1)	0.50	0.571		

COR: crude odds ratio; AOR: adjusted odds ratio; N: frequency.

**Table 2:** Medications used by stroke patients during hospitalization and discharge at Shashemene Referral Hospital, 2012–2017 (N=73).

**Drug used for treatment of stroke**	**Frequency (%)**	**Drugs prescribed on discharge**	**Frequency (%)**
Aspirin 81-325 mg QD	45(61.6)	Aspirin 81 mg QD	34(46.5)
Simvastatin 40 mg QD or Lovastatin 40 mg QD	50(68.5)	Aspirin 81 mg QD & Simvastatin 40 mg QD	24(32.9)
Aspirin 81-325 mg QD & Simvastatin 40 mg QD	25(32.2)	Aspirin 81 mg **Q**D & Lovastatin 40 mg OD	10(13.7)
Aspirin 81-325 mg QD & Lovastatin 40 mg QD	20(27.4)	Lovastatin 40 mg Simvastatin 40 mg	8(10.9) 3(4.1)

QD: once per day.

**Table 3:** Concomitant medication for hospitalized stroke patients at Shashemene Referral Hospital, 2012–2017 (N=73).

**Drug class**	**Drugs**	**Frequency (%)**
Calcium channel blockers	Nifedipine	18(24.7)
	Amlodipine	5(6.8)
Antibiotics	Ceftriaxone	9(12.3)
	Ceftriaxone & Azithromycin	7(9.6)
	Ceftriaxone & Metronidazole	7(9.6)
ACE Inhibitors	Enalapril	15(20.5)
	Captopril	3(4.1)
Diuretics	Furosemide	6(8.2)
	Hydrochlorothiazide	1(1.4)

## Data Availability

The datasets used and/or analyzed during the current study will be shared upon formal permission from the hospital.

## Abbreviations

AOR: Adjusted odds ratio
ART: Antiretroviral therapy
CT: Computed tomography
COR: Crude odds ratio
SPSS: Statistical Package for the Social Sciences
WHO: World health organization.

### Operational Definitions

Stroke: Rapidly developing clinical signs of focal (or global) disturbance of cerebral function, with symptoms lasting 24 h or longer which may lead to death, with no apparent cause other than of vascular origin. It may be diagnosed with imaging or clinical information
Undetermined type of stroke: A type of stroke neither ischemic nor hemorrhagic, not clearly identified
Improved or good outcome: If the patient is discharged without complication
Poor outcome: If the patient is discharged with complication or death.
